# A neonatal mouse model of central nervous system infections caused by Coxsackievirus B5

**DOI:** 10.1038/s41426-018-0186-y

**Published:** 2018-11-21

**Authors:** Qunying Mao, Xiaotian Hao, Yalin Hu, Ruixiao Du, Shuhui Lang, Lianlian Bian, Fan Gao, Ce Yang, Bopei Cui, Fengcai Zhu, Lianzhong Shen, Zhenglun Liang

**Affiliations:** 1 Institute for Biological Products Control, National Institutes for Food and Drug Control, Beijing, China; 2 Quality Control Department, Hualan Biological Engineering Inc., Henan, China; 3 Shandong Xinbo Pharmaceutical Co. Ltd., Dezhou, China; 4 Vaccine Clinical Evaluation Department, Jiangsu Provincial Center for Disease Control and Prevention, Nanjing, China

## Abstract

As one of the key members of the coxsackievirus B group, coxsackievirus B5 (CV-B5) can cause many central nervous system diseases, such as viral encephalitis, aseptic meningitis, and acute flaccid paralysis. Notably, epidemiological data indicate that outbreaks of CV-B5-associated central nervous system (CNS) diseases have been reported worldwide throughout history. In this study, which was conducted to promote CV-B5 vaccine and anti-virus drug research, a 3-day-old BALB/c mouse model was established using a CV-B5 clinical isolate (CV-B5/JS417) as the challenge strain. Mice challenged with CV-B5/JS417 exhibited a series of neural clinical symptoms and death with necrosis of neuronal cells in the cerebral cortex and the entire spinal cord, hindlimb muscles, and cardiomyocytes. The viral load of each tissue at various post-challenge time points suggested that CV-B5 replicated in the small intestine and was subsequently transmitted to various organs via viremia; the virus potentially entered the brain through the spinal axons, causing neuronal cell necrosis. In addition, this mouse model was used to evaluate the protective effect of a CV-B5 vaccine. The results indicated that both the inactivated CV-B5 vaccine and anti-CVB5 serum significantly protected mice from a lethal infection of CV-B5/JS417 by producing neutralizing antibodies. In summary, the first CV-B5 neonatal mouse model has been established and can sustain CNS infections in a manner similar to that observed in humans. This model will be a useful tool for studies on pathogenesis, vaccines, and anti-viral drug evaluations.

## Introduction

Coxsackievirus B5 (CV-B5) belongs to the *Enterovirus* genus in the Picornaviridae family and is a major member of the coxsackievirus B group. CV-B5 is an icosahedral spherical particle with a diameter of 24–30 nm, which consists of a nonenveloped capsid and a core of single-stranded RNA of approximately 7400 nucleotides (nt)^1^. Infections with CV-B5 can cause a variety of central nervous system (CNS) diseases, such as aseptic meningitis^2–5^, viral encephalitis^6–9^, acute flaccid paralysis^10^, myocarditis^11,12^, Type 1 diabetes^13,14^, transient aphasia^15^, herpangina^16^, and hand, foot, and mouth disease (HFMD)^17–19^. Numerous aseptic meningitis and encephalitis outbreaks associated with CV-B5 have been reported worldwide, with clinical characteristics including acute onset, severe clinical symptoms, and high mortality, especially in the last decade^20–30^. This virus is a potential threat to the health of infants and young children. To date, not many data from basic research on CV-B5 infections are available, and currently, there is no effective treatment for the diseases caused by CV-B5. Animal models are the basic tools for pathogenesis research, vaccine development, and drug screening for viral infectious diseases. In this study, we established a neonatal mouse model that exhibited symptoms similar to those observed in human cases, e.g., CNS infections and myocarditis. This mouse model provides a valuable tool for CV-B5-related basic research and vaccine evaluation.

## Results

### Establishment of a CV-B5 susceptible mouse model

**Fig. 1:**
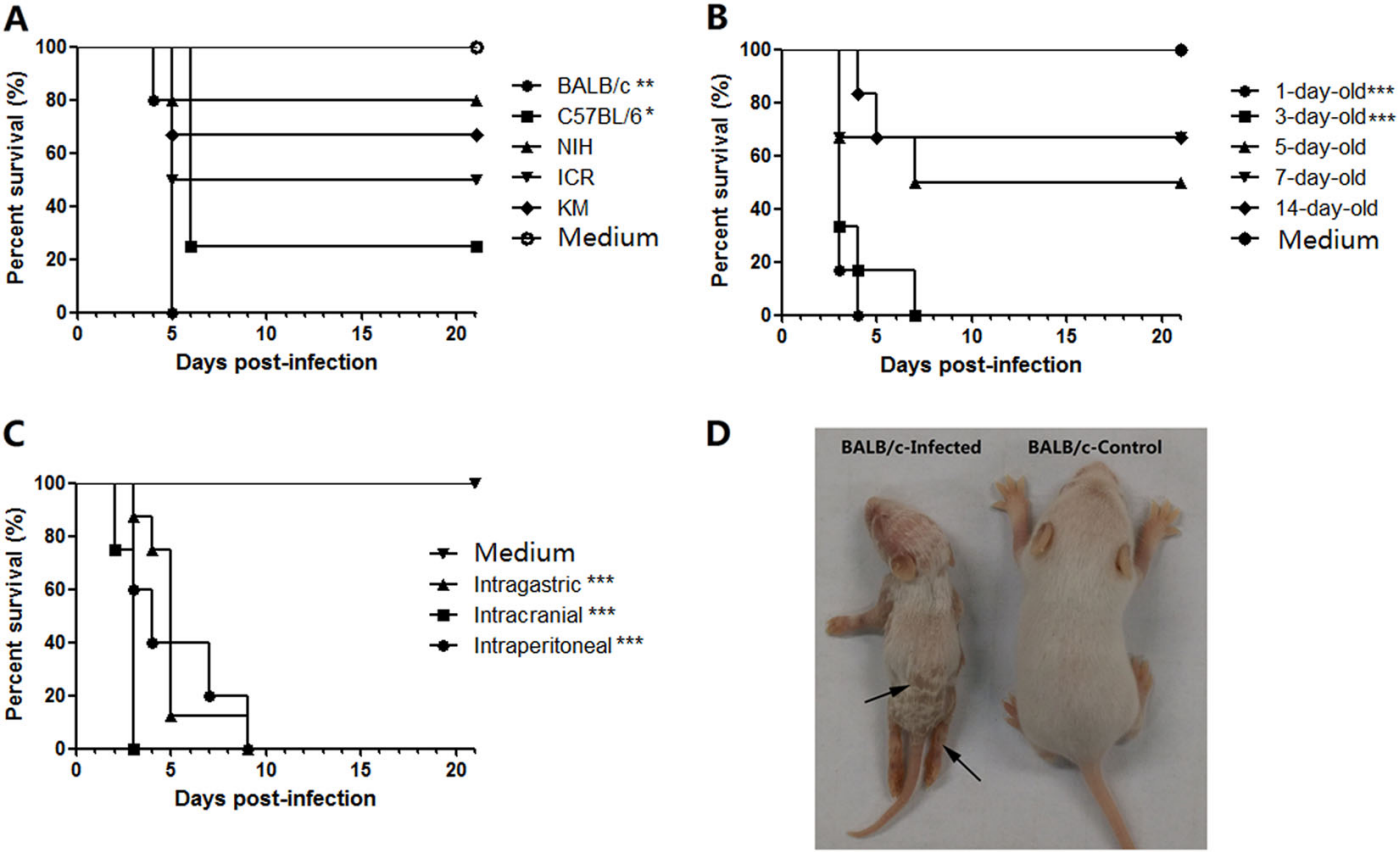
CV-B5 susceptible mouse model with CV-B5/JS417. **a** The process of selecting a suitable mouse strain. One-day-old mice of various strains (BALB/c, C57BL/6, KM, ICR, and NIH) were i.p. challenged with CV-B5/JS417 (3.16 × 10^7^ CCID_50_/mouse). **b** The process of selecting the suitable age. BALB/c mice at 1, 3, 5, 7, and 14 days of age were i.p. challenged with CV-B5/JS417 (3.16 × 10^7^ CCID_50_/mouse). **c** The process for selecting the suitable inoculation route. Three-day-old BALB/c mice were challenged with CV-B5/JS417 (3.16 × 10^7^ CCID_50_/mouse) via the i.p., intracerebral (i.c.), or intragastric (i.g.) route. The control mice were treated with medium via the same corresponding routes. *n* = 6 to 10 mice for each group. All mice were monitored daily for body weight and clinical symptoms for 21 dpi. One representation of two independent experiments was shown. The Mantel–Cox log-rank test was used to compare the survival of the pups between each group and the medium control group at 21 days post-infection. ***p < 0.001. **p < 0.01.* *p* < 0.05 (**d**). A representative image of 3-day-old BALB/c mice infected with CV-B5/JS417 (the left side) or with medium (the right side) at 7 dpi is shown, and the infected mice exhibited the following clinical symptoms: wasting, hair loss, and hindlimb paralysis (arrows)

To select the most susceptible mouse strains, 1-day-old BALB/c, C57BL/6, KM, ICR, and NIH mice were intraperitoneally (i.p.) challenged with CV-B5/JS417 (3.16 × 10^7^ CCID_50_/mouse). All mouse strains developed clinical symptoms, such as inactivity, wasting, shivering, hair loss, hunching, hindlimb paralysis, and even death. The clinical symptoms were ranked from mild to severe, as follows: 0-healthy; 1-inactivity and wasting; 2-shivering; 3-hunched posture and hair loss; 4-hind-limb paralysis; and 5-moribund and death (S1 Table). The survival rates for the BALB/c, C57BL/6, ICR, KM and NIH mice were 0, 25, 50, 67%, and 80% up to 21 days post-infection (dpi), respectively (Fig. 1a). Thus, the BALB/c mouse was the most susceptible strain to CV-B5 infection, and this strain was selected for further study. To assess the susceptibility of mice of various ages to CV-B5 challenge, groups of BALB/c mice aged 1, 3, 5, 7, and 14 days were inoculated (i.p.) with CV-B5/JS417 (3.16 × 10^7^ CCID_50_/mouse). As shown in Fig. 1b, all infected neonatal mice became sick, and some mice died during the experiment. However, the survival rate increased with increasing age. All of the 1-day-old and 3-day-old mice died within 4 dpi and 7 dpi, respectively. The survival rates for 5-, 7-, and 14-day-old mice were 50, 67, and 67% on day 21, respectively. Considering that i.p. administration is more difficult to perform on 1-day-old mice—a difficulty that could potentially result in the administration of a less accurate challenge dose of CV-B5–3-day-old mice were selected for further study.

To select a suitable inoculation route, 3-day-old BALB/c mice were challenged with CV-B5/JS417 via the i.p., intracerebral (i.c.) or intragastric (i.g.) route. As shown in Fig. 1c, all inoculated mice became sick, and the mortality rate was 100%. Mice inoculated via the i.c., i.p., and i.g. route died within 3, 9, and 9 dpi, respectively. Among the three routes of administration, i.g. is most similar to the infection route that occurs in humans. However, this procedure is difficult to perform on a 3-day-old mouse. Therefore, the i.p. route was chosen for further study.

**Fig. 2:**
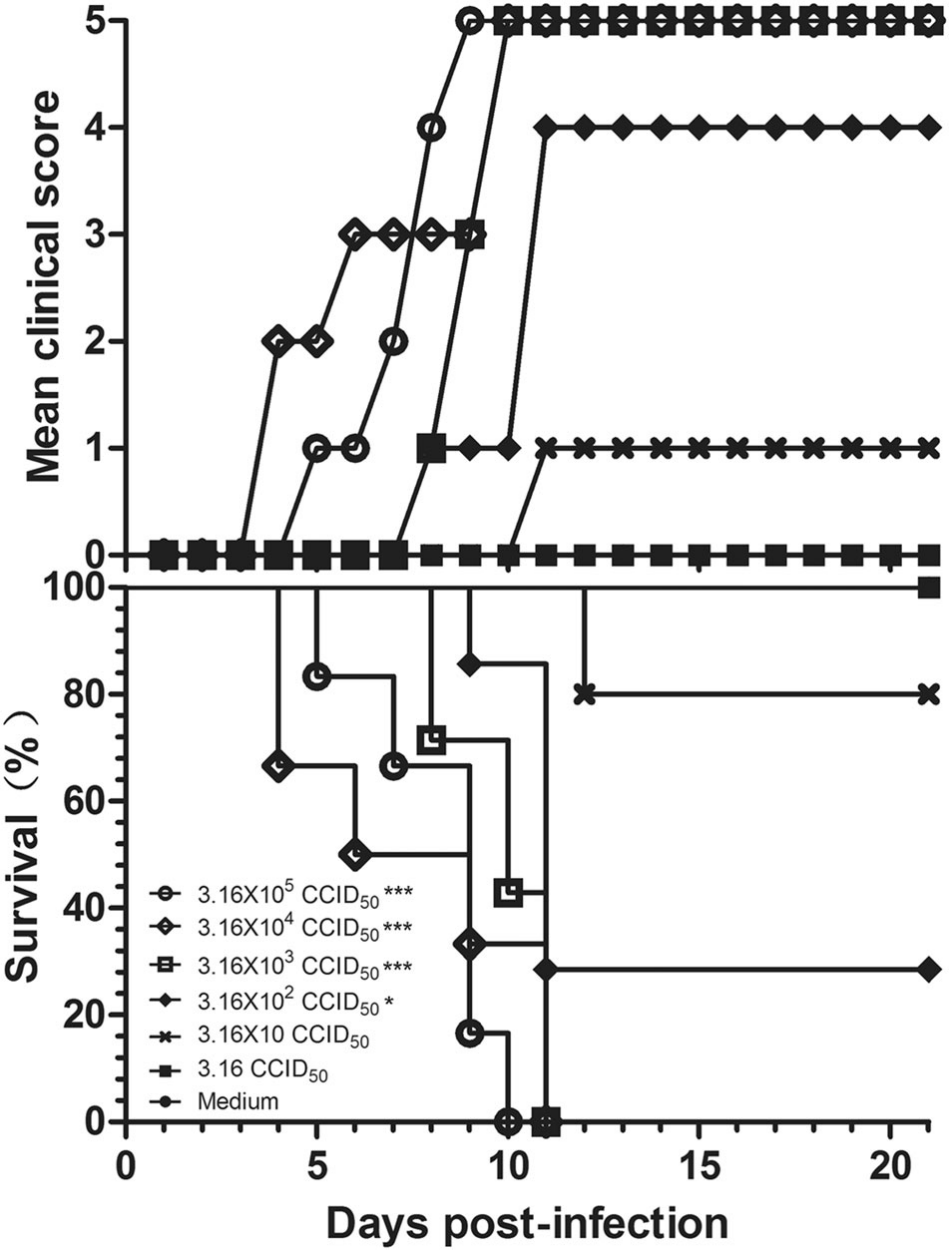
CV-B5 infection in 3-day-old mice resulted in dose-dependent disease and mortality. Three-day-old BALB/c mice (*n* = 6 to 10, per group) were i.p. inoculated with CV-B5/JS417 at a dose ranging from 3.16 to 3.16 × 10^5^ CCID_50_/mouse (10-fold serially diluted). The control mice were treated with medium. The CV-B5/JS417-induced mean score of clinical symptoms and CV-B5/JS417-induced mortality were monitored and recorded daily. Representative results of duplicate experiments are shown. The Mantel–Cox log-rank test was used to compare the survival of the pups between each group and the medium control group at 21 days post-infection. ***p < 0.001. *p < 0.05

To select a suitable challenge dose for the mouse model, 3-day-old BALB/c mice were challenged via the i.p. route with a series of dilutions of CV-B5/JS417 (10-fold dilution from 3.16 × 10^5^ to 3.16 CCID_50_/mouse). A dose-dependent response, including clinical symptom and mortality, was observed (Fig. 2). Mice challenged with CV-B5/JS417 at 3.16 × 10^5^, 3.16 × 10^4^, and 3.16 × 10^3^ CCID_50_/mouse became sick at 4, 3, and 7 dpi, respectively, and a 100% mortality rate was observed at 10, 11, and 11 dpi, respectively. The mice inoculated with 3.16 × 10^2^ and 3.16 × 10 CCID_50_/mouse became sick at 7 and 10 dpi, respectively, and the survival rates were 29% and 80% at 21 dpi, respectively (Fig. 2). In contrast, mice inoculated with 3.16 CCID_50_/mouse remained healthy and exhibited no clinical symptoms up to 21 dpi. The LD_50_ value was 123 CCID_50_/mouse, as calculated by the Reed–Muench method. In consideration of the consistency of clinical symptoms and the mortality rate, a challenge dose of 3.16 × 10^3^ CCID_50_/mouse (26 LD_50_) of CV-B5/JS417 was chosen.

Finally, to investigate the reproducibility of the neonatal mouse model, six replicate experiments were performed on different days under the experimental conditions described in Table 1. Typical clinical symptoms and a 100% mortality rate were consistently observed in all experiments. From three to 5 days post-challenge, the mice began to exhibit disease symptoms, including weight loss, lack of energy, shivering, hunching, hair loss, and hindlimb paralysis (Fig. 1d). In the six independent experiments, death occurred between 4 (4/6) and 8 dpi (2/6). Mortality (100%) occurred between 8 and 11 dpi (Table 1). These results indicated that the challenge with CV-B5/JS417 caused typical clinical symptoms among 3-day-old BALB/c mice and eventually lead to their death. The established experimental conditions showed a good reproducibility. Therefore, this neonatal mouse model was deemed suitable for use as a CV-B5 animal model for drug development and vaccine evaluation.

**Table 1:** Reproducibility of the CV-B5-challenged neonatal mouse model

Experiment #	Onset of symptom (d)	Onset of death (d)	Time of death for all mice (d)	Mortality ratio (%)
1	4	8	8	100
2	3	4	8	100
3	5	8	9	100
4	3	4	9	100
5	3	4	10	100
6	3	4	11	100

Six replicate experiments were performed on different days with three-day-old BALB/c mice (*n* = 6 to 10 per group) that were i.p. challenged by 26 LD50 CV-B5/JS417. CV-B5/JS417-induced clinical symptoms and mortality were monitored and recorded daily

### Pathological changes in mice challenged with CV-B5/JS417

**Fig. 3:**
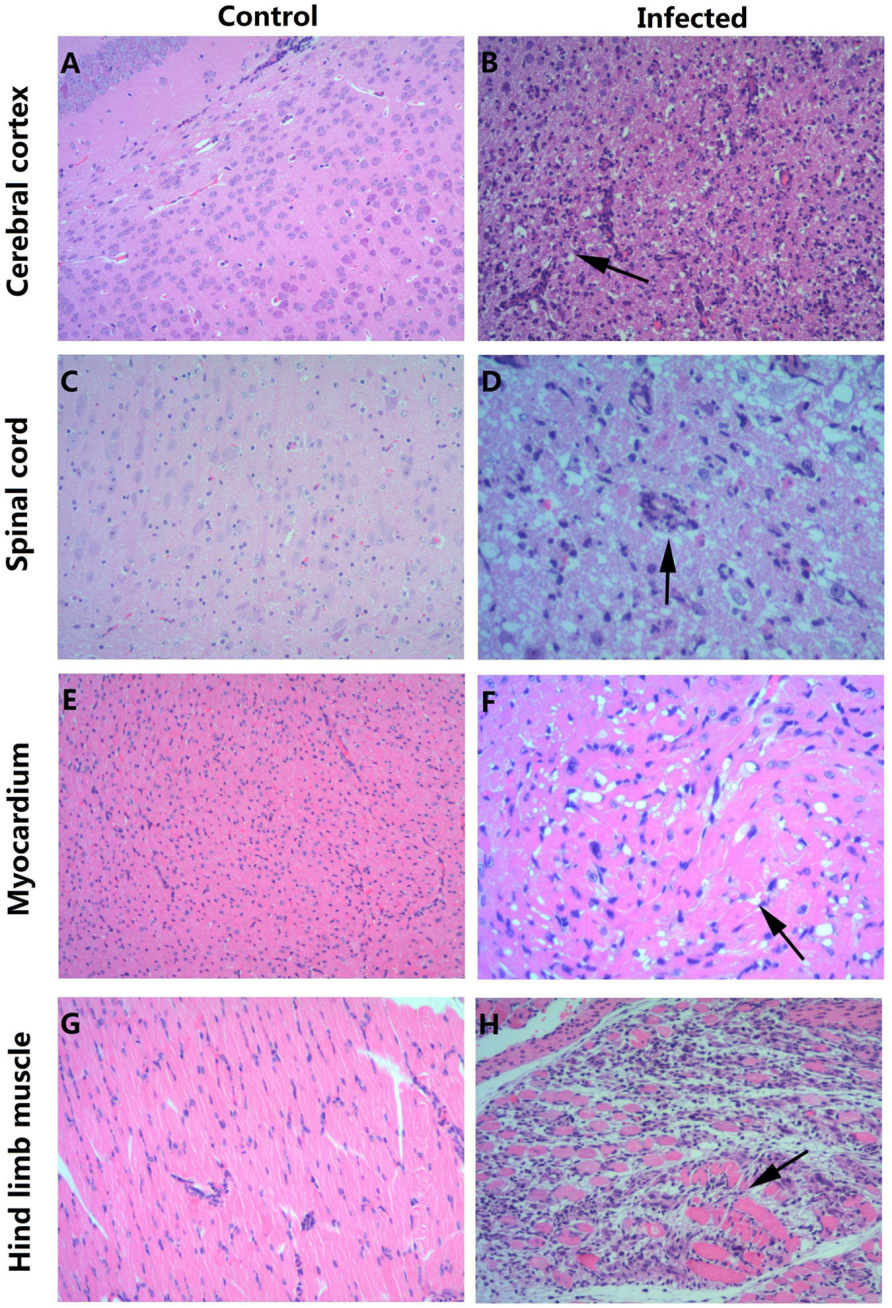
HE staining of various tissues from the BALB/c neonatal mice that were i.p. challenged with CV-B5/JS417. **a**, **c**, **e**, **g** HE staining of cerebral cortex, spinal cord, myocardium, and hindlimb muscle tissues of the control group, respectively. **b**, **d**, **f**, **h** HE staining of corresponding tissues of the experimental group. **b** Necrosis of the cerebral cortex associated with tubular infiltration (arrow); **d**: degeneration and necrosis of spinal cord nerve cells with glial response (arrow); **f**: eosinophilic necrosis of cardiomyocytes (arrow); **h**: necrotic myositis of hindlimb muscle (arrow). Magnifications ×100 (**a**–**c**, **e**, **g**, **h**), Magnifications ×200 (**d**, **f**). *n* = 6–10 mice for each group. One representative image is shown

**Fig. 4:**
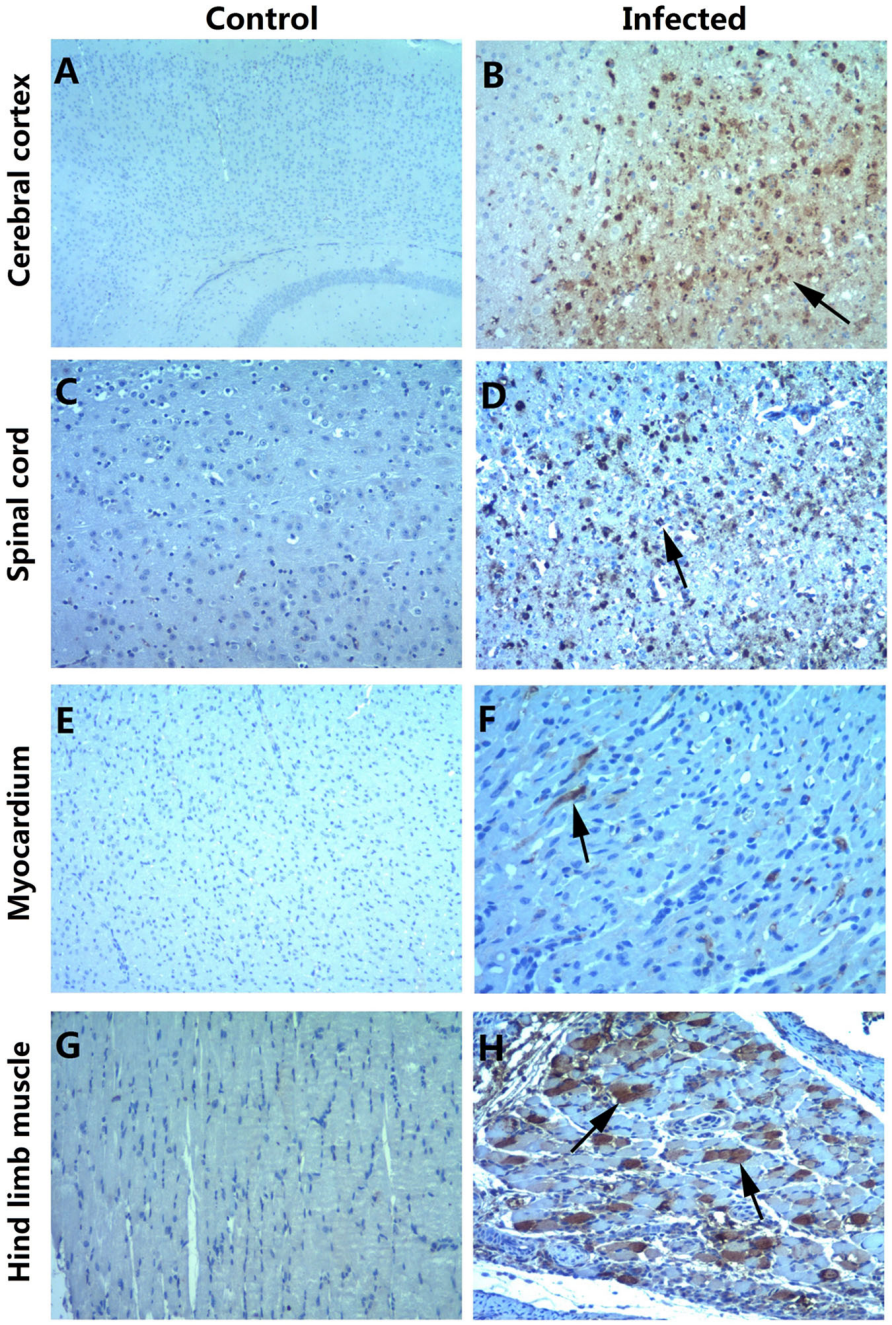
Immunohistochemical staining of various tissues from the BALB/c neonatal mice following the intraperitoneal injection with CV-B5/JS417. **a**, **c**, **e**, **g** IHC staining of the cerebral cortex, spinal cord, myocardium, and hindlimb muscle in the control group, respectively. **b**, **d**, **f**, **h** IHC staining of the cerebral cortex, spinal cord, myocardium, and hindlimb muscle in the experimental group. Positive staining was detected in the cerebral cortex (**b**, arrow), spinal cord (**d**, arrow), myocardium (**f**, arrow) and hindlimb muscle (**h**, arrow) of the neonatal mice after the intraperitoneal injection of CV-B5/JS417. Magnifications ×40 (**a**); magnifications ×100 (**b**–**h**). *n* = 6–10 mice per group. One representative image is shown

To investigate the distribution of the virus and the pathological changes, 3-day-old mice infected with 26 LD_50_ CV-B5/JS417 at Grade Level 5 (S1 Table) were killed for pathological and immunohistochemical (IHC) examinations. The pathological studies revealed that neurons in the cerebral cortex exhibited obvious cell necrosis, gliacyte reaction and infiltration of perivascular infiltrates, eosinophilic necrosis accompanied by gliacyte reaction for the entire spinal cord, eosinophilic necrosis and myositis of hindlimb muscles, and focal eosinophilic necrosis of the cardiomyocytes (Fig. 3). IHC staining revealed strong positivity of the CV-B5 antigen in the cerebral cortex, spinal cord, and hindlimb muscles (Fig. 4). The presence of the CV-B5 antigen was also detected in the myocardium (Fig. 4). These results indicate that CV-B5 can destroy nerve cells and myogenic cells in neonatal mice.

### Viral load (VL) in the tissues of CV-B5/JS417-infected mice

**Fig. 5:**
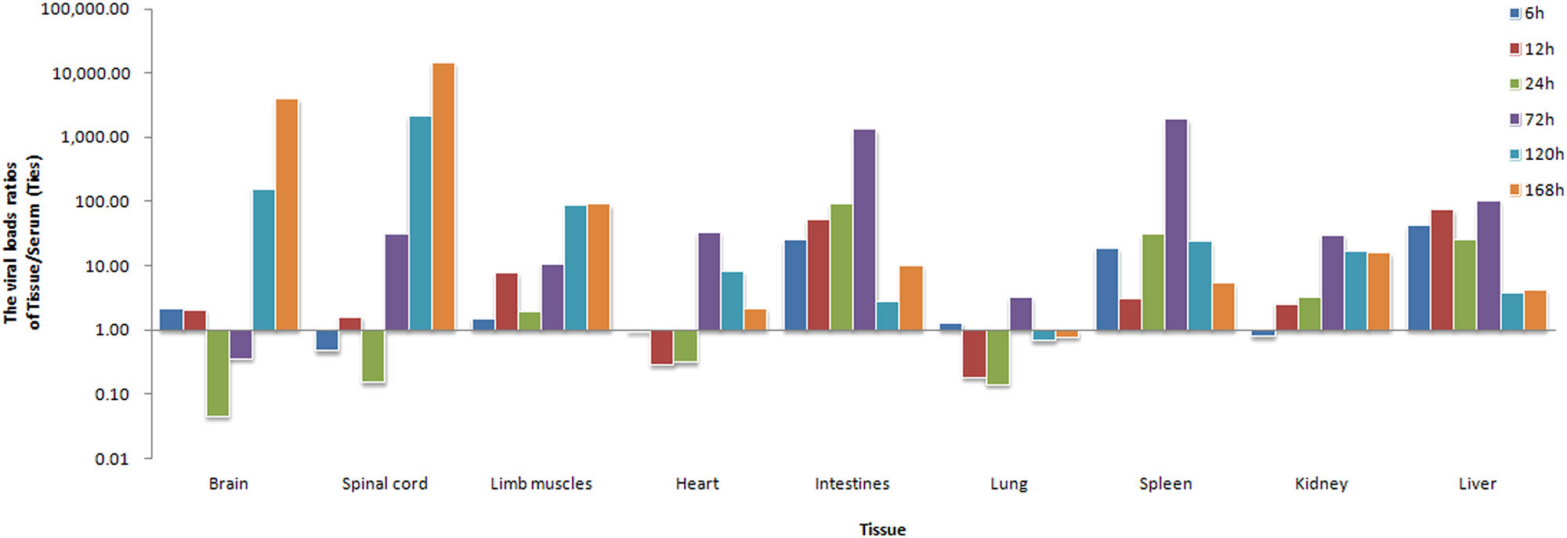
The viral load ratios of tissue/serum in CV-B5-challenged mice. Three-day-old BALB/c mice were i.p. inoculated with CV-B5/JS417 (3.16 × 10^3^ CCID_50_/mouse). To exclude the impact of the viral load in the serum, the viral load ratio (tissue/serum) was calculated by dividing the mean viral load of each tissue with the viral load of the serum at 6, 12, 24, 72, 120, and 168 h post-challenge (*n* = 3, per time point)

To investigate the dynamic changes of CV-B5/JS417 viral loading, blood and tissue samples (heart, liver, spleen, lung, kidney, intestine, brain, spinal cord, and hindlimb muscles) were collected at hour 6, 12, 24, 72, 120, and 168 post-challenge. The VL ratio of tissue/blood at each time point was calculated to exclude the impact of the viral load in the serum. By comparing the VL ratios, it was clear that the viral loading patterns differed among tissue types. These tissues were classified into the following three categories according to the change in VL (Fig. 5):

1. Tissues with high VL (>10) first and then decreasing, included the intestine, spleen and liver, and viral load ratios were 25 to 1318, 19 to 1926, and 43 to 100, respectively, from hour 6 to hour 72 post-challenge; after hour 72, the VL decreased rapidly. The viral load was found to be approximately 1300 times higher in the intestine compared with the blood. Moreover, the VL of the intestine was much higher than the VL found in most other tissues at hour 72. Given the negative results obtained in the pathological examination of these tissues (Figs. 3 and 4), we speculated that the intestine may be the primary target organ of CV-B5 invasion and replication. After the i.p. injection, CV-B5/JS417 may absorb into the bloodstream through the mesentery and rapidly enter the intestinal tissue, leading to rapid viral amplification and replication in the intestine. The viral replication peaked at hour 72 post-injection and a large number of viruses were subsequently released, resulting in the secondary virus infiltration. The change of CV-B5 viral load ratios in the spleen and liver tissues relative to the blood also suggested that the amount of virus in the blood rose continuously until hour 72.
2. Tissues with low VL (<5) first and then increasing, included the spinal cord, brain and hindlimb muscles. The ratios of the viral load in these organs remained at a low level up to hour 24 post-challenge and then increased continuously from hour 72, 120, or 72 till death. At the time of death, the viral load in the brain, spinal cord, and hindlimb muscles was 14,500, 4000, and 90 times higher than that in the blood, respectively, and much higher than the VL found in other organs. Taking into account the results of pathology, histochemistry, and the clinical symptoms, it appeared that the brain, spinal cord and hindlimb muscles may be the privileged target organs. The damage to the brain and spinal cord may be responsible for the CNS clinical manifestations, which eventually lead to CV-B5-associated death.
3. Tissues with low VL (<5) first, then increasing and finally decreasing, included the heart, lung, and kidney. The VL in these tissues was low initially, then peaked at hour 72, and ultimately became low at the end of the experiment. These results suggest that the virus may not persist in these tissues and may increase in the course of the secondary viral invasion in the blood. The pathology and histochemistry results revealed that the cardiomyocyte necrosis resulted from an attack by the high viral load in the blood.

### Active protection from CV-B5 vaccination

**Fig. 6:**
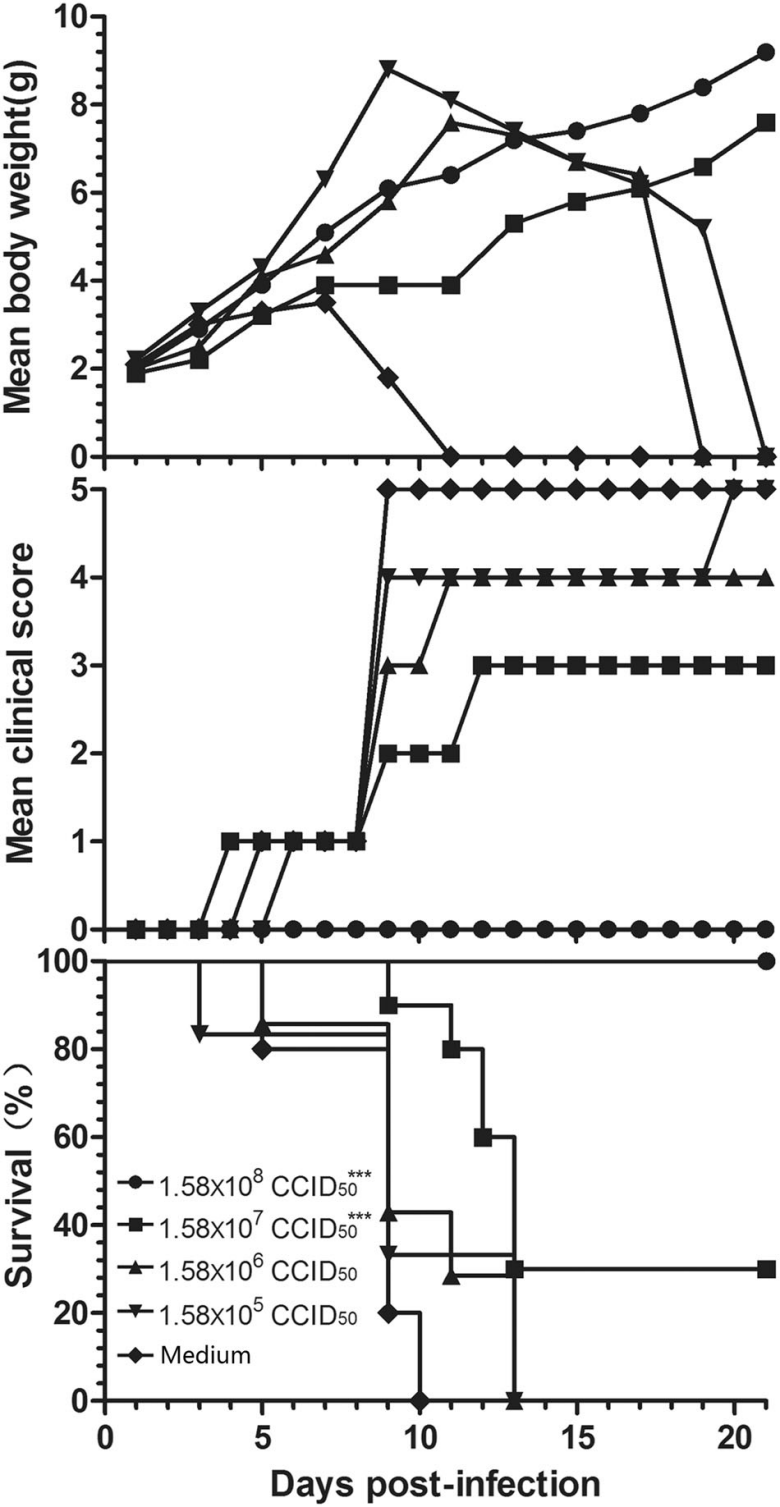
Evaluation of the protective effect of the inactivated CV-B5 vaccine in the mouse model. Adult female BALB/c mice were vaccinated with formaldehyde-inactivated CV-B5/JS417 (experiment group) or medium (control group) twice with a 2-week interval and allowed to mate 1 h after the first vaccination. The resulting pups were challenged with CV-B5/JS417 (3.16 × 10^3^ CCID_50_/mouse) on day 3 after birth. The mortality, clinical symptoms, and body weight were monitored and recorded daily after the infection (*n* = 6 to 10, per group). One representative result is shown. The Mantel–Cox log-rank test was used to compare the survival of the pups between each vaccine group and the medium control group at 21 days post-infection. ***p < 0.001

Female mice (8 weeks old) were immunized with a 10-fold serially diluted, inactivated CV-B5 vaccine at 1.58 × 10^5^ to 1.58 × 10^8^ CCID_50_/mouse, twice, with a 2-week interval. The mice were allowed to mate 1 h after the first immunization. The resulting pups were i.p. challenged with CV-B5/JS417 (26 LD_50_) on day 3 after birth (Fig. 6). The neonatal mice in the control group started to die 5 days after the challenge, and all mice died within 10 days. A dose-dependent protection of the CV-B5 vaccine was observed in the neonatal mice; the survival rates were 100 44%, 30 and 0%, corresponding to the dam mice immunized with a dose of 1.58 × 10^8^, 1.58 × 10^7^, 1.58 × 10^6^, and 1.58 × 10^5^ CCID_50_ CV-B5 vaccine, respectively. All mice born from the 1.58 × 10^8^ CCID_50_-dose dam group exhibited no clinical symptoms during the entire observation period, and they all survived. The Reed–Muench calculation revealed that half of the neonatal mice could have been protected from the lethal challenge if their dams had been immunized with 9.58 × 10^6^ CCID_50_ of the inactivated CV-B5 vaccine.

### Passive protection of anti-CVB5 serum

**Fig. 7:**
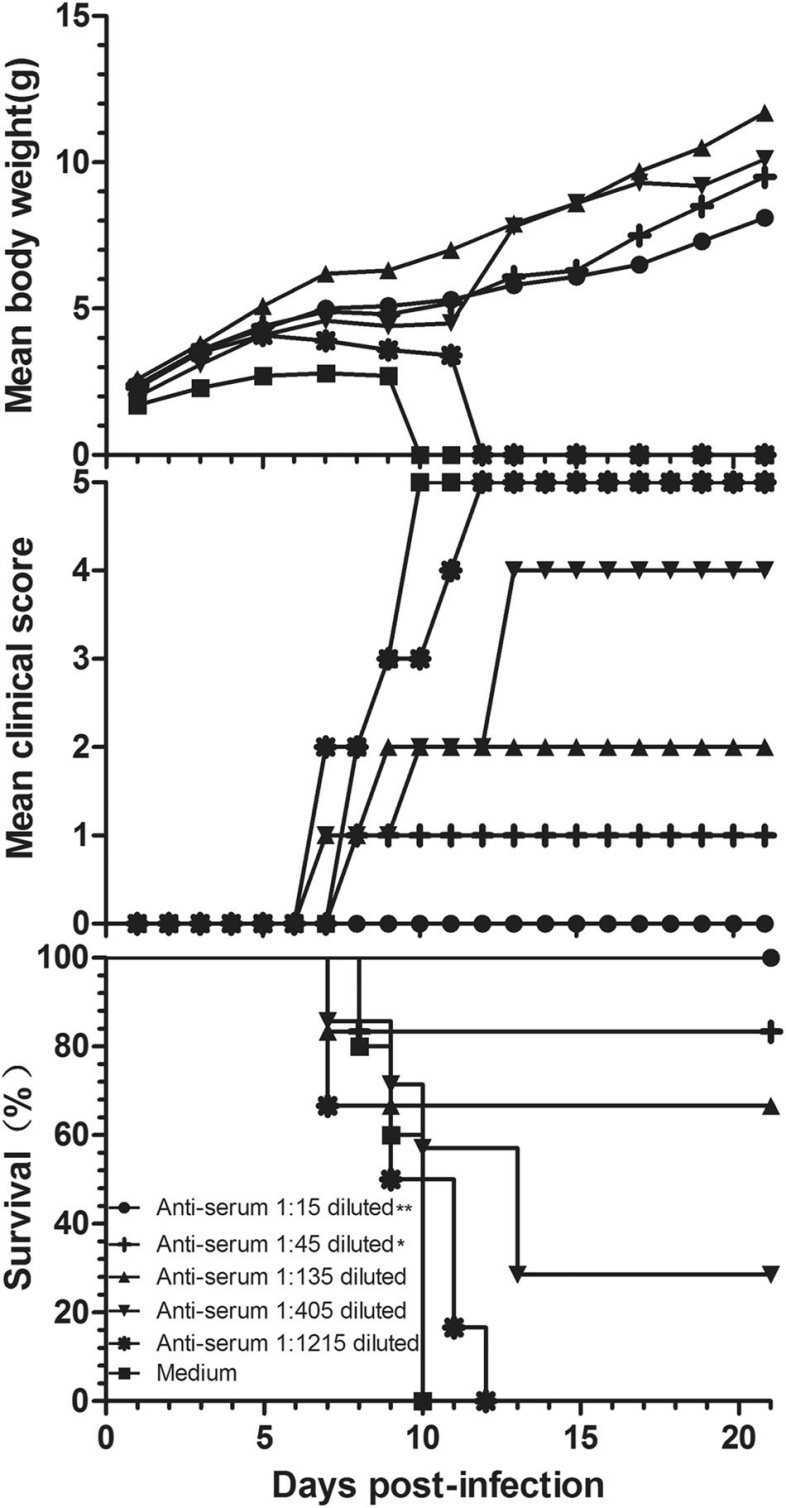
Evaluation of the protective effect of anti-CVB5 serum in the mouse model. Serially diluted anti-CVB5 serum (neutralizing antibody 1:768) or medium was incubated with an equal volume of 3.16 × 10^3^ CCID_50_ CV-B5/JS417 at 37 °C for 1 h. 3-day-old BALB/c mice (*n* = 6–10, per group) were i.p. inoculated with the diluted serum. The mortality, clinical symptoms, and body weight were monitored and recorded daily after the infection. One representative result of two independent experiments is shown. The Mantel–Cox log-rank test was used to compare the survival of the pups between each anti-serum group and the medium control group at 21 days post-infection. **p < 0.01, *p < 0.05

Three-fold serially diluted anti-CVB5 serum (neutralizing antibody 1:768) or medium (control) was incubated with an equal volume of 3.16 × 10^3^ CCID_50_ CV-B5/JS417 at 37 °C for one hour (see Material and Methods). 3-day-old BALB/c mice were i.p. inoculated with the mixture of virus/anti-serum. As shown in Fig. 7, the neonatal mice from the control group started to die on day 8, and 100% mortality was reached within 10 days post-inoculation. The survival rates of the neonatal mice were 100, 83, 67, 29, and 0%, which corresponded to the inoculated mixtures of virus/anti-serum with ratios of 1:15, 1:45, 1:135, 1:405, and 1:1215, respectively. These results indicate that anti-CVB5 serum conferred protection to the experimental animals in a dose-dependent manner. According to the Reed–Muench calculation, the 1:200 dilution of the anti-CVB5 serum protected half of the neonatal mice. The ED_50_ of the neutralizing antibody titer against CV-B5 was 1:3.8.

## Discussion

Animal models are essential tools for virology research. In 2005, C3H/HeJ mice were used to investigate the histopathological changes and the distribution of CV-B5 RNA in the heart, liver, and pancreas during the acute phase of CV-B5 infection^31^. Histological examination revealed CV-B5-induced acute pancreatitis with massive lymphocyte infiltration and loss of acinar cells. However, neither clinical symptoms nor death were reported in that study. In 2009, an in vivo study was conducted on arbidol activity using BALB/c mice challenged with CV-B5^32^. The mice exhibited diminished vitality and weight loss, and they died after the CV-B5 challenge with lung lesions and heart diseases. In addition, several pig models have also been used to study CV-B5^33,34^. However, no CNS infection symptoms, which have been observed in human cases, were reported in these studies.

In the present study, a CV-B5 mouse model was established using 3-day-old BALB/c mice. A clinical strain (CV-B5/JS417) isolated from an HFMD patient in Jiangsu Province of China in 2013 was chosen as the challenge strain, as this strain has shown high toxicity in mice. After the i.p. challenge with CV-B5/JS417 at a dose of 26 LD_50_/mouse, the animals exhibited not only anti-feeding, weight loss, malaise, and hair loss but also a variety of neurological symptoms, such as trembling, hunching, hindlimb paralysis, and even death. The pathological and histochemical examinations revealed eosinophilic necrosis of nerve cells and the gliacyte reaction in the cerebral cortex and the whole spinal cord induced by CV-B5. To our knowledge, this is the first animal model that exhibits severe acute CNS infections induced by CV-B5, thereby resembling the human disease. The degeneration and necrosis of cardiomyocytes were also observed in this model. It is interesting to note that, like the other two main members of coxsackievirus B group, CV-B3 and CV-B4, CV-B5 also causes severe acute pathological lesions in the heart and brain of newborn mice^35–37^. However, CV-B5 infection was not shown to result in pericarditis or chronic autoimmune myocarditis, and these two are the obvious symptoms caused by CV-B4 and CV-B3 in mice^35,38^. In this context, the characteristics of CV-B5 infection are more similar to those of EV71, another enterovirus that can cause aseptic meningitis, encephalitis, and other serious neurological symptoms in infants and young children^39–41^. EV71 has exhibited a wide range of neurotropic cellular properties in both mice and monkeys and has been shown to be distributed throughout the nervous system^42^.

Studying the VL in tissues will hopefully help to clarify the viral translation/distribution in animals. In this study, we found that CV-B5 first appeared in the intestine at hour 6 post-infection and reached its peak at hour 72. However, the VL in the spinal cord, brain and hindlimb muscles remained at low levels up to hour 24 post-challenge, and then the VL continued to increase from hour 72, 120, and 72, respectively. Therefore, CV-B5 may be absorbed into the blood circulation through the mesentery, whereupon it quickly enters and invades the intestinal tissues. After replication (peaking at hour 72) in the intestine, a large amount of virus was released into the blood circulation, resulting in the second wave of blood invasion. Through the blood circulation, the virus entered the spinal cord nerve cells, hindlimb muscles, and myocardial cells, resulting in pathological damage. However, the VL in the brain significantly increased after hour 120, which was later than the increase in VL in the spinal cord and hindlimb muscles. These results suggest that CV-B5 may enter the brain through the spinal axons where it then causes neuronal cell necrosis. These findings are also similar to the mechanism of the EV71 and poliovirus^43–47^. In 2004, the mechanism of EV71 infection was investigated in a 1-day-old mouse model using virus isolation detection. The EV71 antigen was detected in intestinal, thoracic, cervical, and brain stem cells at hour 6, 24, 50 and 78 post-challenge, indicating that EV71 crossed the blood–brain barrier through the axonal transport neural pathway^43^. Further research into the pathway that facilitates CV-B5 transmission to the CNS is warranted.

Vaccines are the most effective and economical means of preventing and controlling infectious diseases. Historically, the development and subsequent application of vaccines for polio and hepatitis A, which are important members of the *Picornaviridae*, have significantly contributed to the prevention and control of polio and hepatitis A on a global scale^48,49^. In recent years, EV71-inactivated vaccines have also shown good safety and immunogenicity with a protective effect of over 90%, providing a powerful weapon in fighting serious HFMD in the Asian-Pacific region^50,51^. To explore the feasibility of a CV-B5 vaccine, in the present study, 8-week-old female mice were immunized with an experimental vaccine from the inactivated CV-B5 virus, and their newborns were challenged with CV-B5/JS417. All of the pups from the control group developed clinical symptoms and eventually died; however, immunization of the female mice with the experimental vaccine effectively protected their newborns from the CV-B5/JS417 challenge. Furthermore, the protection was positively linked to the immunization dosage used for the dams; the newborns exhibited varying responses ranging from no clinical symptoms to delayed onset to death, as the immunization dosage decreased. These results indicate that the inactivated CV-B5 vaccine effectively protected the newborns from the CV-B5 infection in an immunization dose-dependent manner. Immunization of the dams with the inactivated CV-B5 at 9.58 × 10^6^ CCID_50_ protected 50% of the pups from death. Further research on the antibody response in dams and the transfer of antibodies to the pups would be interesting. To further investigate the protective mechanism of the inactivated CV-B5 vaccine, the challenge virus was neutralized with a specific CV-B5 neutralization antibody (titer 1:768), and the antibody/virus mixture was injected into neonatal mice. Dose-dependent protection was again observed. Specific CV-B5 neutralizing antibodies were shown to have an important role in CV-B5 prevention. Epidemiological studies have also indicated that after CV-B5 epidemics, CV-B5 neutralization antibodies in humans increased significantly^52^. These findings lay a solid foundation for the future development of a CV-B5 vaccine.

In summary, a CV-B5/JS417 isolate was successfully used to establish a lethal CV-B5 neonatal mouse model with good reliability and reproducibility; this model exhibited the development of typical CNS infection symptoms similar to those observed in the human disease. This is the first time that an inactivated CV-B5 vaccine has been demonstrated to provide protection against CV-B5 challenge in vivo, in which CV-B5 neutralizing antibodies may have the most important role. This model is a reliable research tool for CV-B5-related research, particularly for research into the development of vaccines and drugs.

## Materials and methods

### Ethics statement

All animal research protocols were approved by the Institutional Animal Care and Use Committee at National Institutes for Food and Drug Control, China (No. 2014-B-004), and these protocols were conducted in accordance with the regulations on the management of laboratory animals (National Science and Technology Commission no. 2 of Oct. 31, 1988) and “guidance notes on the treatment of experimental animals” (Chinese version (2006) no. 398). All institutional guidelines for animal care and use were strictly followed throughout the experiments.

### Cells and viruses

Vero cells (American Type Culture Collection, Manassas, VA) were maintained in Eagle’s minimum essential medium containing 2or 10% fetal bovine serum plus 2 mM l-glutamine, 100 IU/mL penicillin, and 100 μg/mL streptomycin.

The CV-B5 strain CV-B5/JS417 (GenBank: KY303900) used in this study was isolated from a human throat swab sample of a 2-year-old boy, was obtained from a previously described clinical HFMD case collection at Jiangsu Province Center for Disease Control and Prevention and was anonymized in mainland China in 2013^53,54^. The CV-B5/JS417 strain was grown in Vero cells, with a titer of 3.16 × 10^8^ CCID_50_/mL.

### Mouse infection study

The specific pathogen-free (SPF) mice used in this study included inbred C57BL/6, BALB/c, ICR, NIH and Kunming (KM) mice (National Institute for Food and Drug Control, Beijing, China).

First, to establish the CV-B5-susceptible mouse model, the most susceptible mouse strain was identified. 1-day-old BALB/c, C57BL/6, KM, ICR, and NIH mice were i.p. challenged with CV-B5/JS417 (3.16 × 10^7^ CCID_50_/mouse). Second, to determine the more susceptible age, BALB/c mice at 1, 3, 5, 7, and 14 days of age were i.p. challenged with CV-B5/JS417 (3.16 × 10^7^CCID_50_/mouse). Then, to determine the optimal route of administration, 3-day-old BALB/c mice were challenged with CV-B5/JS417 (3.16 × 10^7^ CCID_50_/mouse) via the i.p., i.c., or i.g. route. To determine the optimal dosage of CV-B5, 3-day-old BALB/c mice were i.p. challenged with CV-B5/JS417 at various doses (3.16 to 3.16 × 10^5^ CCID_50_/mouse). The control mice were mock-infected with medium via the same route. Each group contained 6 to 10 mice. All mice were monitored daily for body weight and clinical symptoms until death or 21 dpi. Duplicate experiments were carried out for each experimental condition selection. The LD_50_ was calculated using the method reported by Reed and Muench^55^. Clinical symptoms were ranked from mild to severe, as follows: 0-healthy; 1-inactivity and wasting; 2-shivering; 3-hunched posture and hair loss; 4-hind-limb paralysis; and 5-moribund and death (S1 Table).

### Histopathological examination and immunohistochemical staining (IHC)

Six to ten mice in the experimental group were i.p. challenged with CV-B5/JS417 (3.16 × 10^3^ CCID_50_/mouse). When the clinical symptoms of the challenged mice reached Grade 5 (S1 Table), the mice in both the experimental and control groups were euthanized and subjected to histopathological and immunohistochemical examinations (IHC). The brain, spinal cord, heart, lung, liver, spleen, kidney, intestines, pancreas and hindlimb muscles were isolated, fixed, and embedded in paraffin for the histopathologic examination and immunohistochemical analysis^56^. IHC analysis was performed using the TM HRP-Polymer Anti-Mouse IHC Kit (Fuzhou Maixin Biotechnology Development Co., Ltd., Fuzhou, China) according to the manufacturer’s recommendations. A mouse anti-CVB5 antibody was used as the primary antibody (1:500 dilution) for the immunohistochemical testing group, and the control group was challenged with medium CV-B5/JS417.

### VLs of tissues in post-challenged infant mice

After being i.p. challenged with CV-B5/JS417 (3.16 × 10^3^ CCID_50_/mouse) or uninfected culture medium, tissues and blood samples from the experimental mice (n = 3 per time point) were collected at hour 6, 12, 24, 72, 120, and 168 post-infection to assay the viral loads using real-time qRT-PCR^56^. Blood and homogenized tissues were harvested using the Mag Max^TM^ viral isolation kit (AMBION Inc., Austin, TX, USA). The One Step Prime Script^TM^ RT-PCR Kit (Takara company, Dalian, China) was used for RT-PCR. Briefly, cDNA was synthesized from RNA by reverse transcription for 5 min at 42 °C followed by 10 s at 95 °C. The cDNA was subsequently amplified with 40 cycles of 95 °C for 5 s, and 60 °C for 34 s. The primers used were as follows: forward 5′-CCCAGTGCCTACGAAAGTGA-3′; reverse 5′-CATACGGGGTGGTGCACTC-3′; probe 5′-FAM-TACAGCTGGCAGACGTCCACCAA-BQ1-3′. Viral loads were expressed as log_10_ copies/mg of tissue or log_10_ copies/mL of blood.

### Protective efficacy of the inactivated CV-B5 vaccine

To evaluate the protective efficacy of the CV-B5 vaccine, CV-B5/JS417 (3.16 × 10^8^ CCID_50_/mL) was inactivated with 37% formaldehyde (Sinopharm Group, Beijing, China) at a final concentration of 1/2000 at 37 °C for 3 days. The inactivated viral suspensions were stored at −80 °C. No live virus was detected after the culture was repeated in Vero cells three times. Eight-week-old female BALB/c mice (n = 2 per group) were i.p. injected twice at 2-week intervals with 0.5 mL of 10-fold serially diluted inactivated CV-B5/JS417 or medium alone (as a control). The mice were allowed to mate 1 h after the first injection. After delivery (5 to 10 days after the boost), the pups were i.p. challenged with CV-B5/JS417 (3.16 × 10^3^ CCID_50_/mouse) on day 3 postnatal. The body weight and clinical symptoms of the challenged neonatal mice were monitored daily until death or 21 dpi. The experiments were performed in duplicate. The ED_50_ was calculated based on the method reported by Reed and Muench^55^.

### Protective efficacy of neutralizing antibodies

To understand the protective efficacy of the CV-B5 neutralizing antibody, neonatal mice were passively immunized to protect them against the CV-B5/JS417 challenge using mouse anti-CVB5 serum (neutralizing antibody 1:768). The anti-CVB5 serum was 3-fold serially diluted from 1:15 to 1:1215 and then mixed with an equal volume of CV-B5/JS417 virus (3.16 × 10^3^ CCID_50_/mouse). Medium that was treated in the same manner, rather than anti-serum, was used as the control. After inoculation at 37 ℃ for 1 h in a tube, 3-day-old BALB/c mice (*n* = 6 to 10 per group) were i.p. inoculated with serially diluted mixtures. The mortality and clinical symptoms were then monitored and recorded daily until 21 dpi. The experiments were performed in duplicate. The ED_50_ was calculated based on the method reported by Reed and Muench^55^.

### Statistical analysis

All statistical analyses were performed with GraphPad Prism software (version 5.01) for Windows. Survival curves were compared by the log-rank (Mantel–Cox) test. The 50% lethal dose (LD_50_) and the dose required for 50% of the maximal effect (ED_50_) were determined by the methods described by Reed and Muench^55^. A *p* value of <0.05 was considered statistically significant.

## Electronic supplementary material

S1 Table
