# Supplementary material for: A neonatal mouse model of central nervous system infections caused by Coxsackievirus B5
Source: Emerg Microbes Infect. 2018 Nov 21;7:185. doi: 10.1038/s41426-018-0186-y (PMC6246558; doi:10.1038/s41426-018-0186-y)
Supplement: Supplementary file 1 — S1 Table [file 41426_2018_186_MOESM1_ESM.pdf]

S1 Table Grades for the clinical symptoms of CV-B5/JS417-infected mice

Grade	Clinical signs
0	Healthy
1	Inactivity / Wasting
2	Shiver
3	Hunching / Hair loss
4	Hind limb paralysis
5	Moribund / Death
